# Zuranolone for treatment of major depressive disorder: a systematic review and meta-analysis

**DOI:** 10.3389/fnins.2024.1361692

**Published:** 2024-04-25

**Authors:** Abdullah Ahmad, Abdul Rafeh Awan, Natasha Nadeem, Aamir Shahid Javed, Mobeen Farooqi, Muhammed Daniyal, Hassan Mumtaz

**Affiliations:** ^1^CMH Lahore Medical College, National University of Medical Sciences, Lahore, Pakistan; ^2^Department of Medicine, Nishtar Medical University, Multan, Pakistan; ^3^Department of Data Analytics, BPP University, London, United Kingdom

**Keywords:** Zuranolone, major depressive disorder, depression, postpartum depression, antidepressants, essential tremors

## Abstract

**Background:**

Current treatment modalities for Major Depressive Disorder have variable efficacies and a variety of side effects. To amend this, many trials for short term, well tolerated monotherapies are underway. One such option is Zuranolone (SAGE-217), which is a recent FDA approved antidepressant for *Post Partum* depression (PPD) and is undergoing clinical trials for PPD, major depressive disorder (MDD) and essential tremors (ET).

**Objectives:**

Pool currently available data that compare Zuranolone to Placebo for the treatment of Major Depressive Disorder and evaluate its efficacy and safety profile.

**Methods:**

We retrieved data from PUBMED and SCOPUS from inception to July 2023. We included articles comparing Zuranolone or SAGE 217 with placebo in patients suffering from Major Depressive Disorder. Review Manager 5.4 was used to analyze the outcomes including changes in the Hamilton Depression Rating Scale (HAM-D), Hamilton Anxiety Rating Scale (HAM-A) and Montgomery–Åsberg Depression Rating Scale (MADRS) scores from baseline as well as any treatment emergent adverse events (TEAEs) and severe adverse events.

**Results:**

Our review analyzed 4 trials and the data of 1,357 patients. Patients treated with Zuranolone indicated a statistically significant effect in the change from baseline in HAM-D score (*p* = 0.0009; MD [95% CI]: −2.03 [−3.23, −0.84]) as well as in MADRS score (*p* = 0.02; MD [95% CI]: −2.30[−4.31, −0.30]) and HAM-A score (*p* = 0.03; MD [95% CI]: −1.41[−2.70, −0.11]) on 15th day when compared to the Placebo group. Zuranolone was also significantly associated with a higher response rate (*p* = 0.0008; OR [95% CI]: 1.63[1.14, 2.35]) and higher remission rate (*p* = 0.03; OR [95% CI]: 1.65[1.05, 2.59]) when compared with the placebo. As for safety, Zuranolone was significantly associated with 1 or more TEAE (*p* = 0.006; RR [95% CI]: 1.14[1.04, 1.24]) but an insignificant association with side effects that lead to drug discontinuation (*p* = 0.70; RR [95% CI]: 1.18[0.51, 2.76]) and serious adverse events (*p* = 0.48; RR [95% CI]: 1.46 [0.52, 4.10]) when compared with placebo.

**Conclusion:**

Zuranolone is an effective and safe drug for short course major depressive disorder monotherapy. It shows results in 14 days (compared to 2–4 weeks that SSRI’s take) and has anti-anxiolytic effects as well. However, only 4 trials have been used for the analysis and the sample size was small. The trials reviewed also cannot determine the long-term effects of the drug. More trials are needed to determine long term effects.

## 1 Introduction

Approximately 280 million people in the world have depression, which amounts to nearly 3.8% of the population (Vos et al., 2020). Major Depressive Disorder (MDD) is a mood disorder which has been identified as the largest contributor to global disability (Rehm and Shield, 2019) and is associated with significant comorbidities as well as a greater risk of mortality from all causes compared to nondepressed individuals (Kozela et al., 2016). The etiology of MDD is complex, with environmental, genetic and epigenetic factors playing a role in its pathophysiology. The most accepted theories include the depletion of neurotransmitters such as monoamines, gamma-aminobutyric acid (GABA), and brain-derived neurotrophic factor (BDNP), disturbances in the hypothalamic–pituitary–adrenal axis, inflammation, and structural and functional brain changes (Thom et al., 2019).

However, standard-of-care (SOC) antidepressants are associated with treatment-limiting adverse effects including cognitive impairment, arrhythmias, falls, fractures, seizures, suicidal thoughts, hyponatremia, weight changes, and sleep disturbances (Sobieraj et al., 2019). Further, despite Selective Serotonin Reuptake Inhibitors (SSRIs) remaining the most widely-used drug for the treatment of MDD, efficacy has been found to be incomplete with 60–70% of patients not achieving remission, and 30–40% not showing a significant response to treatment (Yuan et al., 2020). It has been recommended that a short-term course of monotherapy which is well-tolerated would lead to a paradigm shift in the treatment of MDD (Clayton et al., 2023a).

Zuranolone (SAGE-217) is a neuroactive steroid and a positive allosteric modulator of γ-aminobutyric acid A (GABA_A_) receptors which has been investigated in clinical trials for the treatment of postpartum depression (PPD), MDD, and essential tremor (ET) (Martinez Botella et al., 2017). One study found that zuranolone had the third best surface under the cumulative ranking curve (SUCRA) ranking (58.8%) for the treatment of postpartum depression (Clayton et al., 2023a). Due to its drug metabolic and pharmacokinetic (DMPK) profile, zuranolone has been formulated as an oral, once-daily course for 14 days for the treatment of MDD (Center for Behavioral Health Statistics, 2019; Hoffmann et al., 2020). Zuranolone has demonstrated synergistic effects with diazepam and enhances GABA receptor activity through increased cell surface expression as opposed to benzodiazepines (Althaus et al., 2020). Clinical trials have shown improvement in depressive symptoms and a well-tolerated course of zuranolone in patients with MDD (Zhang et al., 2022). Given the growing need for a reduction in the global burden of MDD with tolerable monotherapies, we sought to evaluate the efficacy and safety of zuranolone for the treatment of MDD in comparison to placebo as a primary outcome of this meta-analysis.

## 2 Methodology

This review article was conducted in conformity with Cochrane (Higgins et al., 2024) and PRISMA (Page et al., 2021) guidelines.

### 2.1 Search strategy and selection of articles

A search of PubMed, SCOPUS and Google scholar was conducted on July 8th, 2023 from inception to July 2023. No filters were applied, and articles of every language were considered in our initial search. The following mesh terms were used in our search string: Revi ((zuranolone) OR (SAGE-217)) AND ((MDD) OR (major depressive disorder) OR (depression)).

### 2.2 Eligibility criteria

#### 2.2.1 Inclusion criteria

1. Randomized control trials evaluating the efficacy and safety of zuranolone for patients with major depressive disorder (MDD) as the primary or secondary outcome.
2. No restriction to age, gender, language or country was applied.
3. Only original studies were used.

#### 2.2.2 Exclusion criteria

1. Non-randomized controlled trials, case reports, case series, observational studies, commentaries, review articles were excluded.
2. Studies focusing on the use of drug in patients suffering from postpartum depression.
3. Non-human or animal studies trials
4. Only peer-reviewed publications were considered.

### 2.3 Data extraction

The data from the selected studies including study characteristics, baseline demographics and outcome data were extracted onto a predefined Excel sheet. The data extraction was performed by two independent reviewers (ARA, AA) and any discrepancy was settled by mutual discussion. No assumptions were made regarding any missing data.

### 2.4 Quality assessment

The Cochrane Risk of Bias Tool (ROB II) was used to assess the risk of bias in the included studies. The assessment was carried out independently by two investigators (MF, MD) on multiple aspects: selection of result, outcome measurement, missing outcome data, deviations from intervention, randomization process, and overall bias. Disagreement on the quality of studies was low and was resolved by authors’ consensus.

### 2.5 Data analysis

Review Manager (RevMan Version 5.4; The Cochrane Collaboration, 2020) was used for the statistical analysis. The total number of events and total patients were extracted from each study and the mean difference with 95% CI was then calculated by the software. A random effect model was used and a *p*-value of <0.05 was taken to be statistically significant. Heterogeneity was assessed using the I^2^ statistic (Higgins et al., 2003). Subgroup analyses were performed based on dosage of drug given to the patients. Cochrane risk of bias tool was used to assess the risk of bias among the studies (Higgins et al., 2011). Publication bias could not be assessed as only 4 studies were chosen for inclusion.

## 3 Results

### 3.1 Summary of search

**Figure 1:**
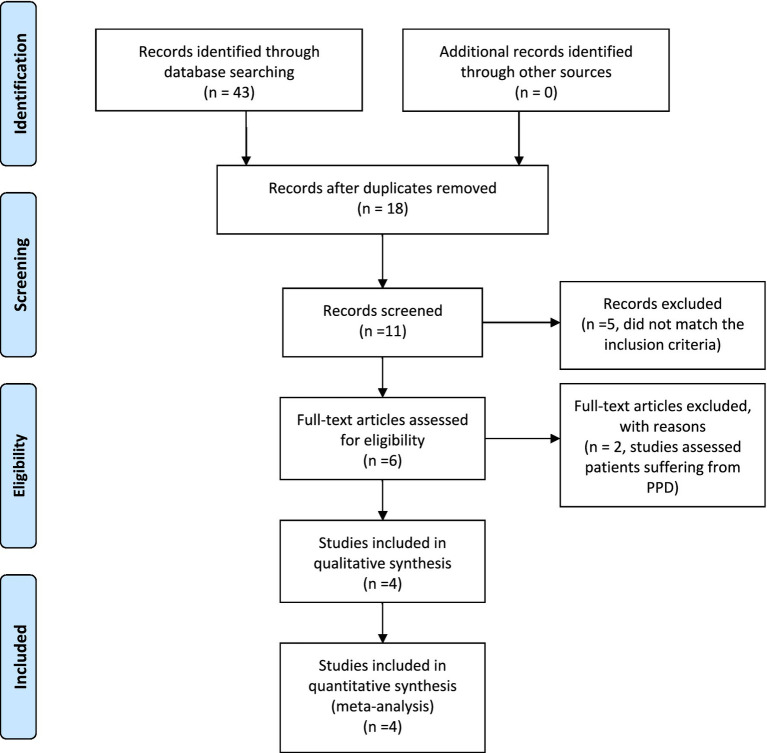
PRISMA flow diagram. Adapted from Moher et al. (2009).

Our search string yielded 43 results which were manually screened to remove duplicates which left us with a total of 11 articles. These 11 articles were then screened independently by 2 reviewers (AA, ARA.) based on their title, abstract and complete text. Of these 11 articles, 6 articles were preliminarily selected as they fit our search strategy (refer to Figure 1). However, on further review of the text, two articles were excluded due to inclusion of postpartum depression patients. Hence, a total of 4 articles were finalized to be included in our review. All arising discrepancies were settled by discussion and mutual consensus among reviewers.

### 3.2 Study characteristics

As per our inclusion criteria, four randomized control trials were included in our review. Three of the trials took place in the United States (Gunduz-Bruce et al., MOUNTAIN trail, Clayton et al.) (Gunduz-Bruce et al., 2019; Clayton et al., 2023a,b) while one trial took place in Japan (Kato et al.) (Kato et al., 2023). Two trials (Clayton et al., Gunduz-Bruce H et al.) (Gunduz-Bruce et al., 2019; Clayton et al., 2023b) lasted 6 weeks. The total duration of Kato et al. (2023) was 14 weeks while the MOUNTAIN trial (Clayton et al., 2023a) lasted for 6 months. The trials took place in the period between 2017 and 2023.

### 3.3 Participant baseline characteristics

As per our inclusion criteria, four randomized control trials and 1,357 people were included in our review (*n* = 808 for the zuranolone arms regardless of dose and *n* = 552 for the placebo group). Majority of the participants were female (65.51%) while 50.63% of the population was white, followed by African Americans (26.46%). A total of 571 people were assessed for the use of antidepressants at baseline, majority (57.44%) of which fit that description. A total of 338 people were evaluated for history of Previous Depressive episode during baseline assessment and most of participants (68.6%) confirmed such episodes. Every participant was subjected to a 14-day treatment period (refer to Table 1).

**Table 1:** Baseline characteristics table.

Baseline characteristics											
	Handan Gunduz et al.; 2017		MOUNTAIN trial; 2023			Anita Clayton et al.; 2023		Masaki Kato et al.; 2023			Combined values
	SAGE-217	Placebo	Zuranalone 30 mg	Zuranalone 20 mg	Placebo	Zuranalone 50 mg	Placebo	Zuranalone 30 mg	Zuranalone 20 mg	Placebo	
Total participants	45	44	166	159	157	268	269	85	82	82	1,357
Males	20	14	45	47	51	82	103	36	35	35	468 (34.49%)
Asian	1	0	2	3	3	13	4	85	82	82	275 (20.27%)
Black	36	28	64	56	54	75	46	0	0	0	359 (26.46)
White	7	16	94	99	96	169	206	0	0	0	687 (50.63%)
Hispanic	0	0	27	31	26	58	54	0	0	0	196 (14.44%)
Age (years)	49.1 ± 13.6	38.3 ± 12.2	42.3 ± 11.8	41.9 ± 12.2	41.4 ± 12.2	39.4 ± 12.3	40.1 ± 12.6	39.3 ± 12.6	38.8 ± 12.0	40.8 ± 10.6	0.74 [−1.66, 3.14]
BMI (kg/m^2^)	30.0 ± 6.3	29.9 ± 5.2	–	–	–	29.6 ± 6.3	30.3 ± 6.2	23.9 ± 4.4	22.7 ± 4.0	23.6 ± 5.3	−0.45 [−1.16, 0.25]
Use of antidepressants at baseline	12	10	47	46	49	–	–	–	–	–	328 (57.44%)
Previous depressive episodes	43	40	–	–	–	–	–	53	44	46	226 (68.6%)
Mean HAM-D score at start of trial	25.2 ± 2.6	25.7 ± 2.4	25.9 ± 2.9	25.8 ± 2.8	25.8 ± 3.1	26.8 ± 2.6	26.9 ± 2.7	24.9 ± 2.4	24.6 ± 2.2	24.5 ± 2.1	-
Days for drug administration	14	14	14	14	14	14	14	14	14	14	14 days

### 3.4 Efficacy outcomes

#### 3.4.1 Analysis of primary efficacy outcomes

**Figure 2:**
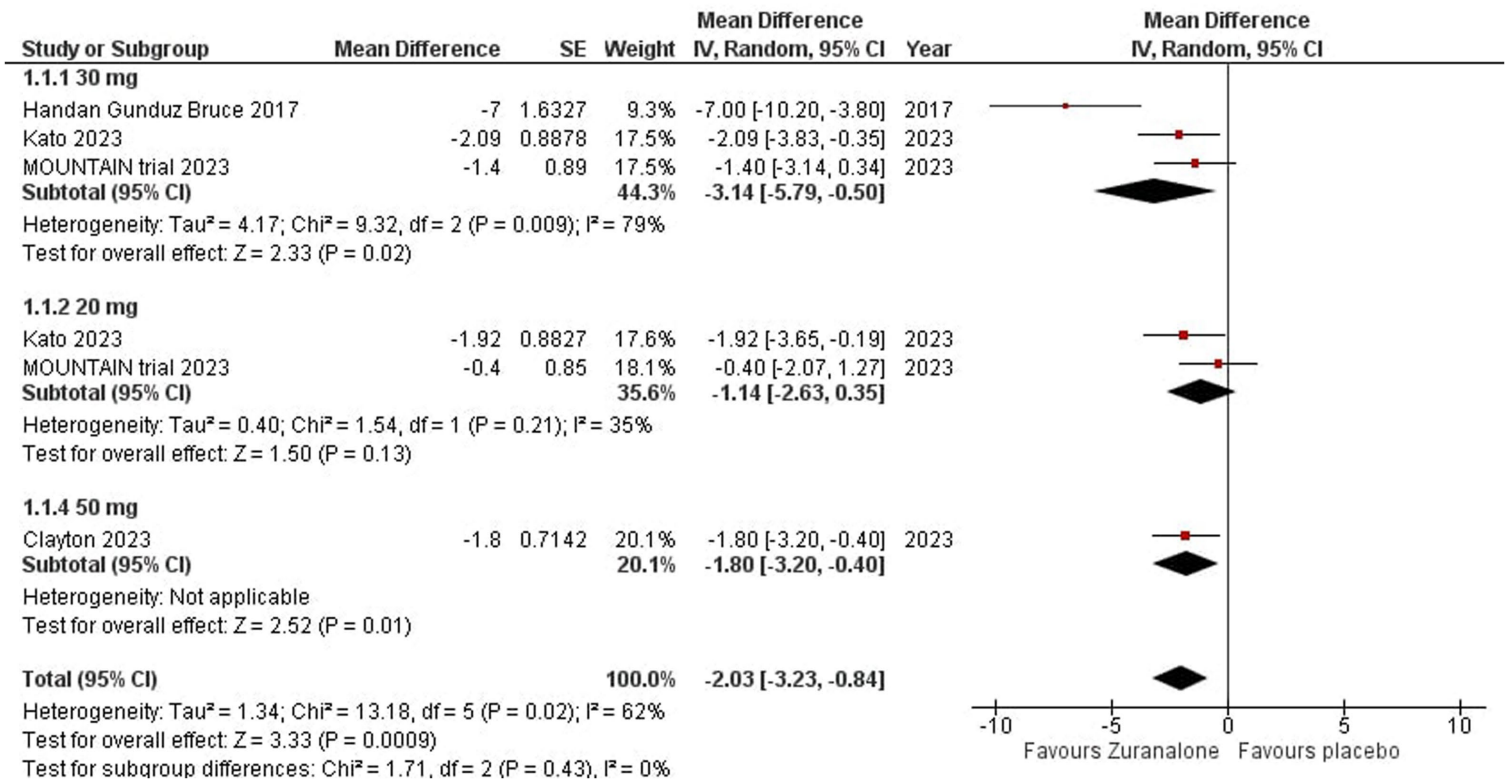
Forest plot for change from baseline (CFB) of HAM-D at day 15.

All our studies reported the change from baseline (CFB) of the Hamilton Depression Rating Scale (HAM-D) at Day 15 (Gunduz-Bruce et al., 2019; Kato et al., 2023; Clayton et al., 2023a,b). Overall, zuranolone group showed a statistically significant difference from baseline in HAM-D scores at day 15 compared to placebo group (*p* = 0.0009; MD [95% CI]: −2.03 [−3.23, −0.84]; I^2^ = 62%). Our sub group analysis (divided based on the dose of zuranolone given to patients) showed that 30 mg zuranolone had the most significant difference in baseline compared to placebo group (*p* = 0.02; MD [95% CI]: −3.14[−5.79, −0.50]; I^2^ = 79%), followed by 50 mg zuranolone (*p* = 0.01; MD [95% CI]: −1.80[−3.20, −0.40]) 0.20 mg dose showed an insignificant difference (*p* = 0.13; MD [95% CI]: −1.14[−2.63, 0.35]; I^2^ = 35%). Sensitivity analysis was done to identify the cause of high heterogeneity. Gunduz-Bruce et al. (2019) was identified as the outlier (elimination of which reduced the heterogeneity to 0%). Our subgroup analysis (divided based on the region zuranolone was given to patients) showed that US based studies had a more significant difference in baseline compared to placebo group (*p* = 0.02; MD [95%CI]: −2.99[−5.50, −0.47]; I^2^ = 80%) compared to Japanese studies (*p* = 0.02; MD [95%CI]: −2.09[−3.83, −0.35]). Refer to Figure 2 for the forest plot based sub-grouped based on dosage.

#### 3.4.2 Analysis of secondary efficacy outcomes

**Figure 3:**
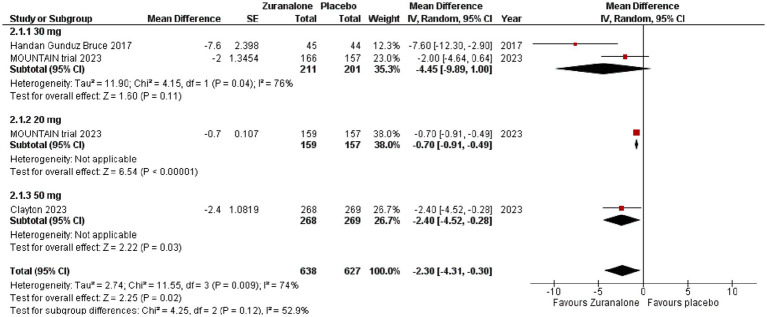
Forest plot for CFB of MADRS at day 15.

Three of our studies reported CFB of the Montgomery–Åsberg Depression Rating Scale (MADRS) and Hamilton Anxiety Rating Scale (HAM-A) at day 15 (total *n* = 1,108, zuranolone *n* = 638, placebo *n* = 470) (Gunduz-Bruce et al., 2019; Clayton et al., 2023a,b). Pooling of data showed that zuranolone had a significant effect on MADRS score compared to placebo (*p* = 0.02; MD [95% CI]: −2.30[−4.31, −0.30]; I^2^ = 74%). After making subgroups based on dose, 50 mg of zuranolone showed a significant difference vs. placebo (*p* = 0.03; MD [95% CI]: −2.40[−4.52, −0.28]). Although, 20 mg of zuranolone had the least difference, it was still statistically significant (*p* < 0.0001; MD [95% CI]: −0.70[−0.91, −0.49]) 0.30 mg of zuranolone showed the highest difference but it was statistically insignificant (*p* = 0.11; MD [95% CI]: −4.45[−9.89, 1.00]; I^2^ = 76%). Refer to Figure 3 for the forest plot.

**Figure 4:**
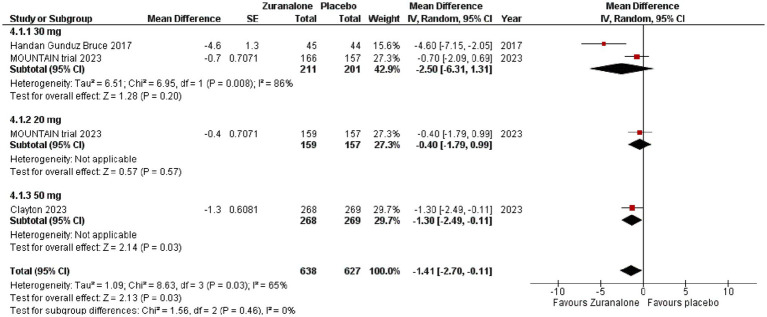
Forest plot for CFB of HAM-A at day 15.

For the CFB of HAM-A score, pooling of data showed that zuranolone again had significant difference compared to placebo (*p* = 0.03; MD [95% CI]: −1.41[−2.70, −0.11]; I^2^ = 65%). Subgroup analysis showed that only 50 mg of zuranolone had a significant difference (*p* = 0.03; MD [95% CI]: −1.30[−2.49, −0.11]). In tandem with CFB in MADRS, 30 mg had the greatest difference, but the results were statistically insignificant (*p* = 0.20; MD [95% CI]: −2.50 [−6.31, 1.31]). 20 mg of zuranolone had a statistically insignificant effect on the CFB of HAM-A (*p* = 0.57; MD [95% CI]: −0.40[−1.79, 0.99]). Refer to Figure 4 for the forest plot.

Three of our studies reported the CFB of HAM-D at day 3 and day 8 (Total *n* = 1,024, zuranolone *n* = 516 and placebo *n* = 508) (Kato et al., 2023; Clayton et al., 2023a,b). Zuranolone had a statistically significant mean difference in the baseline of HAM-D at day 3 (*p* = 0.03; MD [95% CI]: −1.69[−2.61, −0.78]; I^2^ = 65%). Subgroup analysis showed that 50 mg zuranolone had the higher difference versus placebo (*p* < 0.00001; MD [95% CI]: −3.00[−3.06, −2.94]) compared to 30 mg zuranolone versus placebo (*p* < 0.00001; MD [95% CI]: −1.39[−1.80, −0.98]; I^2^ = 97%) both of which are significant. Twenty milligram zuranolone had the least difference but it was significant (*p* = 0.05; MD [95%CI]: −1.02[−2.02, −0.02]).

Consistent with our previous results, zuranolone had a statistically significant mean difference in the CFB of HAM-D at day 8 as well (*p* < 0.00001; MD [95% CI]: −2.12[−2.83, −1.43]; I^2^ = 0%). Subgroup analysis showed that 30 mg zuranolone (*p* < 0.00001; MD [95% CI]: −2.09[−3.15, −1.03]; I^2^ = 0%) had a lower mean difference in baseline as compared to 50 mg zuranolone (*p* < 0.00001; MD [95% CI]: −2.50[−3.75, −1.25]) 0.20 mg zuranolone had the least (*p* = 0.02; MD [95% CI]: −1.69[−3.12, −0.26]). All values were significant.

All of our studies reported HAM-D response rates (proportion of patients who showed a > 50% reduction in HAM-D score) and remission rates (proportion of patients who showed a HAM-D score of 7 or lower) at day 15 (Gunduz-Bruce et al., 2019; Kato et al., 2023; Clayton et al., 2023a,b). In comparison to placebo groups, zuranolone was significantly associated with a higher response rate (*p* = 0.0008; OR [95% CI]: 1.63[1.14, 2.35]; I^2^ = 61%) and higher remission rate (*p* = 0.03; OR [95% CI]: 1.65[1.05, 2.59]; I^2^ = 61%). Sub group analysis showed that 50 mg zuranolone had a significant association with higher response rate (p = 0.03; OR [95% CI]:1.44[1.03, 2.03]) but insignificant association with higher remission rate (*p* = 0.049;OR [95% CI]:1.14 [0.79, 1.66]). Thirty milligram had a significant association with both higher response rate (*p* = 0.03;OR [95% CI]:2.46[1.09, 5.54]; I^2^ = 76%) and higher remission rate (*p* = 0.04;OR [95% CI]:2.56[1.05, 6.24]; I^2^ = 67%) 0.20 mg showed insignificant association with both remission (*p* = 0.59;OR [95% CI]:1.11[0.76, 1.64]; I^2^ = 47%) and response rate (*p* = 0.62;OR [95% CI]:1.34[0.53, 3.37]; I^2^ = 47%).

Sub group analysis based on regions showed that Japanese trials showed a higher remission rate (*p* = 0.91;OR[95% CI]; 2.60[0.98, 6.87]; I^2^ = 0%) compared to the US trials (*p* = 0.01; OR[95%CI] = 1.53[0.91, 2.57]; I^2^ = 73%) but both were not significant. Similarly for response rate, Japanese trials showed a higher rate (*p* = 0.39; OR[95%CI]; 1.96[1.14, 3.37]; I^2^ = 0%) compared to US trials (*p* = 0.01; OD[95%CI]; 1.56[0.99, 2.48]; I^2^ = 72%), however, only Japanese trials results have a significant difference.

Only 2 out of four included studies assessed CFB of Bech 6 test at day 15 (total *n* = 338, zuranolone *n* = 212, placebo *n* = 126) (Clayton et al., 2023a; Kato et al., 2023). Zuranolone demonstrated an insignificant difference in Bech 6 scores (*p* = 0.11; MD [95% CI]: −5.48 [−12.15, 1.20]; I^2^ = 76%) compared to placebo. Zuranolone demonstrated a significant difference in Bech 6 scores in US based population (*p* = 0.0003; MD [95%CI]: −15.10 [−23.30, −6.90]) whereas it showed an insignificant response in Japanese studies (*p* = 0.24; MD [95%CI]: −1.97[−5.28, 1.33]; I^2^ = 0%).

Sensitivity analysis was employed to identify the cause of high heterogeneity for all the secondary outcomes. Removing Gunduz-Bruce et al. (2019) from CFB of HAM-A, HAM-D response rate, HAM-D remission rate and CFB of Bech 6 drops the heterogeneity to 0 and 40% in CFB for MADRS. Sensitivity analysis could not find the cause of the very high heterogeneity of CFB of HAM-D at day 3.

### 3.5 Safety outcomes

**Figure 5:**
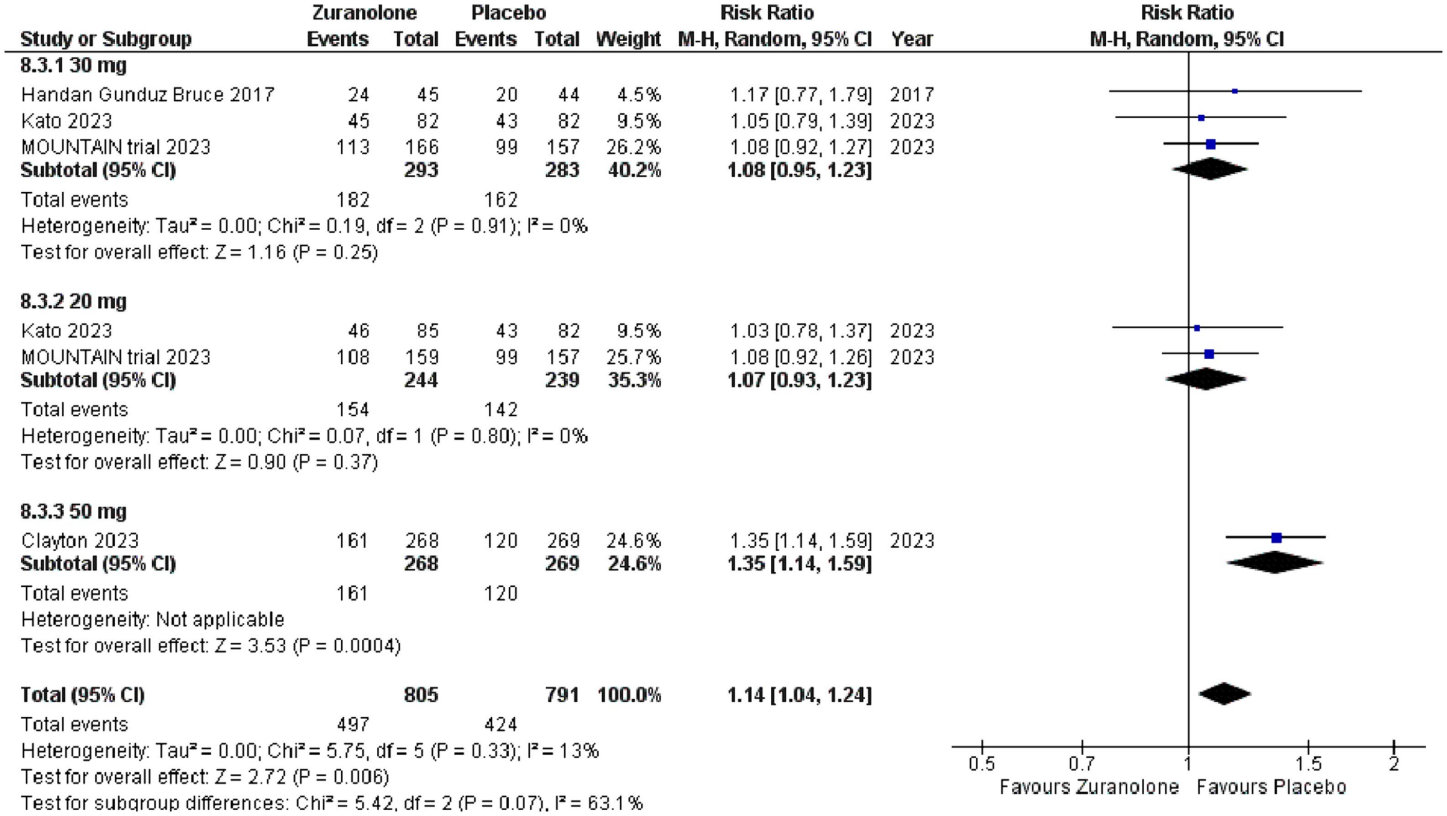
Forest plot for patients who suffered from at least 1 TEAE.

All 4 of our included studies presented data of participants who suffered at least 1 treatment-emergent adverse event (TEAE), participants who suffered any TEAEs that lead to the discontinuation of the drug and participants who suffered from serious Adverse Events (SAE). For people who suffered at least one TEAE, zuranolone had a statistically significant association compared to placebo group (*p* = 0.006; RR [95% CI]:1.14[1.04, 1.24]; I^2^ = 13%). Subgroup analysis determined that only 50 mg zuranolone was significantly associated with at least 1 TEAE (*p* = 0.0004;RR [95% CI]:1.35[1.14, 1.59]). While the 30 mg and 20 mg were also associated with TEAE, the results were not significant. Following were the most experienced TEAEs by the participants; headache (9.29%), somnolence (8.62%), dizziness (7.52%), sedation (3.83%). Refer to Figure 5 for the forest plot. Subgroup analysis determined that US based studies showed zuranolone was significantly associated with at least 1 TEAE (*p* = 0.0005; RR [95% CI]:1.50[1.19, 1.88]) whereas the Japan based studies associated with TEAE, the results were not significant.

Zuranolone was associated with more Serious Adverse effects (SAE) compared to the placebo group however the difference was not statistically significant (*p* = 0.48; RR [95% CI]:1.46 [0.52, 4.10]; I^2^ = 0%). This trend continued when assessing for TEAEs that lead to the discontinuation of the drug, where zuranolone was more associated with such TEAEs but the difference was insignificant (*p* = 0.70; RR [95% CI]: 1.18[0.51, 2.76]; I^2^ = 26%).

### 3.6 Risk of bias assessment

The studies overall had moderate to good methodological quality (Supplementary Figure S1). All the studies showed adequate randomization, as well as patient follow up and outcome reporting. Two of the studies reported relatively more patients withdrew from the study, while all other parameters were completely acceptable. Detailed quality assessment is given in Supplementary Table S1.

## 4 Discussion

Our results show that zuranolone meets its efficacy and safety outcomes. Zuranolone group had a significant mean difference from the baseline values of HAM-D scale, compared to baseline, on day 15 as well as day 3 and day 8. Zuranolone showed a similar response with the CFB of MADRS scale, HAM-A scale, Remission rates and Response rate. On the safety side, zuranolone was significantly associated with at least 1 TEAEs but its association with SAEs and TEAEs that lead to drug discontinuation were insignificant.

Our results found that zuranolone had a significant CFB in HAM-D scores at day 15 as compared to the placebo group (*p* = 0.0009). This is consistent and more significant than the CFB in HAMD-17 results of a previous clinical trial with a two-week course of 30 mg of zuranolone for PPD though it is to be noted that this trial considered CFB of HAMD-17 scores higher than 26 and demonstrated a higher mean difference (SMD = −2.03 for zuranolone in MDD vs. SMD = −4.2 for zuranolone in PPD) (Deligiannidis et al., 2021). Furthermore, the significant difference observed in reduction of depressive symptoms by zuranolone was higher than another drug, curcumin, reviewed for use in MDD (*p* = 0.0009 vs. *p* = 0.002; SMD = −2.03 vs. −0.34) (Al-Karawi et al., 2016), which also demonstrated a non-significant reduction in depressive symptoms with a short-course administration of less than 6 weeks.

Three of the four reviewed clinical trials demonstrated a statistically significant difference, highest in 50 mg of zuranolone, in CFB from HAMD-17 at day 8, which evidences the rapid onset of zuranolone and is consistent with previous studies (Arnaud et al., 2021; Clayton et al., 2022). The rapid response of zuranolone is due to its activity as a NAS at GABA_A_ receptors which occurs within minutes as compared to the slow-onset action of steroid hormones (Joëls, 1997). The rapid onset was concluded to be higher compared with SOC antidepressants like SSRIs, SNRIs, mirtazapine, and vortioxetine (Posternak and Zimmerman, 2005; Kato et al., 2023).

Upon considering, our results indicate a significant difference between zuranolone and placebo based on MADRS scores. Specifically, both 50 and 20 mg doses of zuranolone showed significant differences, while the 30 mg dose of zuranolone demonstrated the highest difference, yet it was not statistically significant. The observed differences in HAM-D and MADRS scores between the 30 mg and 50/20 mg doses of zuranolone were likely due to the smaller sample sizes in these four clinical trials. Conducting further trials with larger sample sizes can provide a clearer picture of zuranolone’s potent antidepressant effects compared to other investigational drugs.

Our meta-analysis also showed a statistically significant associated for patients suffering from at least one TEAE compared to placebo (*p* = 0.006; RR = 1.14), with only 50 mg of zuranolone being significantly associated as opposed to the insignificant results of 30 mg and 20 mg of zuranolone suggesting safer outcomes of the lower doses. Region based subgroup analysis determined that only US based studies showed a significant difference in at least 1 TEAEs whereas, Japan studies showed insignificant difference. TEAEs did lead to the discontinuation of the drug, however, this difference was not significant (*p* = 0.70, RR = 1.18). Further, although zuranolone was associated with more SAE compared to placebo, this difference was not significant (*p* = 0.48, RR = 1.46). These results are positive and are consistent with earlier studies which suggest that the adverse effects are temporary, dose-dependent, and mild (Hoffmann et al., 2020; Sonoyama et al., 2023).

Zuranolone has demonstrated anxiolytic effects (Gauthier and Nuss, 2015) due to its action on different binding sites than benzodiazepines and modulation of GABAA receptors with both phasic and tonic inhibition (Laverty et al., 2017; Martinez Botella et al., 2017). As a short-course monotherapy, it can replace benzodiazepines in combination with antidepressants, reducing the risk of tolerance and abuse (Schmitz, 2016; Wilbraham et al., 2020). Our meta-analysis showed significant improvements in HAM-A scores, particularly with the 50 mg dose of zuranolone. Patients with MDD and elevated anxiety experienced better outcomes with zuranolone compared to the placebo group. This highlights the potential of zuranolone in treating MDD patients with anxiety, warranting further exploration of long-term outcomes and investigating its dose-dependent therapeutic effects as an antidepressant, anxiolytic, and anti-convulsant (Althaus et al., 2020).

Although, zuranolone’s long-term effects, and its role in mild and moderate depression remain uncertain, its short-course and rapid response rate have notable clinical implications. Zuranolone’s rapid response can effectively alleviate major depressive symptoms, making it valuable in psychiatric emergencies, particularly for suicidal ideation (Scala et al., 2023). This can improve patient satisfaction with symptomatic relief and increase their receptivity to future long-term treatment with standard-of-care (SOC) antidepressants. Additionally, a clinical trial showed that patients co-initiated with zuranolone, and a SOC antidepressant experienced better outcome compared to those co-initiated with placebo and a SOC antidepressant (ClinicalTrials.gov, 2024), reinforcing the potential benefits of using zuranolone alongside SOC antidepressant therapy.

To our knowledge, this meta-analysis is the first to explore the efficacy of zuranolone as a treatment option for MDD only and the first to focus on its short term effects. The only other meta-analyze (to our knowledge) has mostly focused on zuranolone as a broad-spectrum treatment option for MDD and its subtypes like PPD (Li et al., 2024). However, though PPD has a high correlation to MDD, etiologically, it has unique genetic components (Guintivano et al., 2023). In fact, Zuranolone was recently approved by the FDA as the first oral treatment for postpartum depression only (U.S. Food and Drug Administration, 2023). Its not yet approved for MDD by FDA. This further underscores that MDD is a heterogenous category and all types of depression cannot be classified within a single category and treated indiscriminately with the same medication without considering the duration and severity of those disorders (Paris, 2014).

### 4.1 Limitations

The results reported in this study has certain limitations due to strict inclusion and exclusion criteria, and due to the inclusion of only four studies (with only the data of 1,357 participants pooled for analysis). The trials focused on a specific subset of patients with Major Depressive Disorder (MDD), and the limited observation points at days 3, 8, and 15 restrict the scope of the study in not analyzing the long-term efficacy and safety. Further, though sub-regional analysis was conducted, there remains a limitation in the influence of regional and cultural differences between three studies that were conducted in the US, and one which was carried out in Japan.

The study is also confined in its analysis of dose differences as it lacks true comparability due to only one study using 50 mg dose, only two studies using 20 mg dose. and only three studies using 30 mg dose. Expanding the number of trials would establish a more robust understanding of zuranolone’s therapeutic effects across different doses, determine the optimal dosage, and assess potential secondary effects and side-effects more comprehensively.

Additionally, to broaden applicability, future trials should encompass non-Major Depressive Disorders, Drug-Resistant MDD, and non-one-dimensional depressive disorders, providing a more comprehensive understanding of zuranolone’s therapeutic potential.

Zuranolone showed significant sustained antidepressant effects through Day 45 in previous PPD studies (Deligiannidis et al., 2021). However, in the trials assessed here, only one study demonstrated improvements in depressive and anxiety symptoms through day 42. Other trials had mixed findings, and there was insufficient data for this meta-analysis to comment on the scope of Zuranolone’s sustained effect. Further clinical trials are needed to investigate the efficacy of Zuranolone beyond 2 weeks (Kato et al., 2023).

## Data availability statement

The raw data supporting the conclusions of this article will be made available by the authors, without undue reservation.

## Author contributions

AAh: Conceptualization, Data curation, Formal analysis, Writing – original draft. AAw: Data curation, Formal analysis, Investigation, Methodology, Writing – original draft. NN: Conceptualization, Data curation, Formal analysis, Investigation, Methodology, Writing – original draft. AJ: Data curation, Formal analysis, Investigation, Project administration, Writing – original draft. MF: Data curation, Formal analysis, Investigation, Methodology, Validation, Writing – review & editing. MD: Data curation, Formal analysis, Investigation, Methodology, Project administration, Visualization, Writing – review & editing. HM: Conceptualization, Methodology, Project administration, Visualization, Writing – review & editing.
