# Supplementary material for: Zuranolone for treatment of major depressive disorder: a systematic review and meta-analysis
Source: Front Neurosci. 2024 Apr 25;18:1361692. doi: 10.3389/fnins.2024.1361692 (PMC11079210; doi:10.3389/fnins.2024.1361692)
Supplement: Supplementary file 1 [file Data_Sheet_1.docx]

**Risk of Bias : ZURANOLONE v Placebo**

Fig 1 (Supplementary)

Table 1 (supplementary)

| No | Study | Experimental | Control | Outcome | Weight | D1 | D2 | D3 | D4 | D5 | Overall |
| --- | --- | --- | --- | --- | --- | --- | --- | --- | --- | --- | --- |
| 1 | Clayton et al | Zuranolone | Placebo | HAMA-D (day 15) | 1 | + | + | ! | + | + | + |
| 2 | Clayton et al | Zuranolone | Placebo | HAMA-D (day 15) | 1 | + | + | ! | + | + | + |
| 3 | Kato et al | Zuranolone | Placebo | HAMA-D (day 15) | 1 | + | + | + | + | + | + |
| 4 | Hamdan et al | Zuranolone | Placebo | HAMA-D (day 15) | 1 | + | + | + | + | + | + |
| D1: Randomisation Process  D2: Deviations from the intended interventions  D3: Missing Outcomes data  D4: Measurement of the outcome  D5: Selection of the reported result  Key = + : Low risk, ! : Some concerns, - : High risk | | | | | | | | | | | |
